# Involvement of Salicylic Acid in Anthracnose Infection in Tea Plants Revealed by Transcriptome Profiling

**DOI:** 10.3390/ijms20102439

**Published:** 2019-05-17

**Authors:** Yun-Long Shi, Yue-Yue Sheng, Zhuo-Yu Cai, Rui Yang, Qing-Sheng Li, Xu-Min Li, Da Li, Xiao-Yuan Guo, Jian-Liang Lu, Jian-Hui Ye, Kai-Rong Wang, Long-Jie Zhang, Yue-Rong Liang, Xin-Qiang Zheng

**Affiliations:** 1 Tea Research Institute, Zhejiang University, # 866 Yuhangtang Road, Hangzhou 310058, China; 11516051@zju.edu.cn (Y.-L.S.); 21816156@zju.edu.cn (Y.-Y.S.); 21716160@zju.edu.cn (Z.-Y.C.); kexiaonao@163.com (R.Y.); qsli@zju.edu.cn (Q.-S.L.); 21616096@zju.edu.cn (X.-M.L.); 11816014@zju.edu.cn (D.L.); GRACE_XY@126.com (X.-Y.G.); jllu@zju.edu.cn (J.-L.L.); jianhuiye@zju.edu.cn (J.-H.Y.); 2 Ningbo Huangjinyun Tea Science and Technology Co. Ltd., Yuyao 315412, China; wkrtea321hjytea@163.com (K.-R.W.); zhanglongjie0701@163.com (L.-J.Z.)

**Keywords:** anthracnose, tea plant, salicylic acid, *Camellia sinensis*, plant immunity, RNA sequencing, PR1, plant-pathogen interaction

## Abstract

Anthracnose is a major leaf disease in tea plant induced by *Colletotrichum*, which has led to substantial losses in yield and quality of tea. The molecular mechanism with regards to responses or resistance to anthracnose in tea remains unclear. A de novo transcriptome assembly dataset was generated from healthy and anthracnose-infected leaves on tea cultivars “Longjing-43” (LJ43) and “Zhenong-139” (ZN139), with 381.52 million pair-end reads, encompassing 47.78 billion bases. The unigenes were annotated versus Gene Ontology (GO), Kyoto Encyclopedia of Genes and Genomes (KEGG), National Center for Biotechnology Information (NCBI) non-redundant protein sequences (Nr), evolutionary genealogy of genes: Non-supervised Orthologous Groups (eggNOG) and Swiss-prot. The number of differential expression genes (DEGs) detected between healthy and infected leaves was 1621 in LJ43 and 3089 in ZN139. The GO and KEGG enrichment analysis revealed that the DEGs were highly enriched in catalytic activity, oxidation-reduction, cell-wall reinforcement, plant hormone signal transduction and plant-pathogen interaction. Further studies by quantitative reverse transcriptase polymerase chain reaction (qRT-PCR) and high-performance liquid chromatography (HPLC) showed that expression of genes involved in endogenous salicylic acid biosynthesis and also accumulation of foliar salicylic acid are involved in the response of tea plant to anthracnose infection. This study firstly provided novel insight in salicylic acid acting as a key compound in the responses of tea plant to anthracnose disease. The transcriptome dataset in this study will facilitate to profile gene expression and metabolic networks associated with tea plant immunity against anthracnose.

## 1. Introduction

Anthracnose is a key leaf disease in the tea plant (*Camellia sinensis* (L.) O. Kuntze) induced by *Colletotrichums* [1,2], which has caused a great loss in tea yield and quality owing to its strong infectivity and widespread distribution [3]. Although there have been hundreds of *Colletotrichum* isolates detected in tea plant [4,5], the dominant species or major pathogen on the tea plant are considered to be *Colletotrichum gloeosporioidesis*, *C. camelliae* and *C. fructicola* [3,4,5], which grow and spread quickly in humid and hot summer and autumn seasons, leading to gray sunken or shrunk necrotic lesions on tea leaves and twigs [6,7]. The loss of tea yield induced by anthracnose was estimated to range from 5% to 20% or even more, depending on the cultivar planted [8,9]. Although plant activators [10], antagonistic bacteria [11] and agronomic techniques such as leaf trimming and rational fertilization have been used to control the anthracnose disease, spraying fungicides is still a major control method in tea fields, resulting in a serious fungicides residue problem. Breeding tea cultivars resistant to anthracnose is considered to be the most effective measure to control anthracnose because there is a great difference in resistance to this disease between various tea cultivars [6,8,9].

Revealing molecular defense responses of tea plant to anthracnose infection and developing a molecular assisted selection (MAS) method are badly needed in the tea plant breeding field. However, little is known about the molecular mechanisms regulating the defense response in tea plants [6] although attempts were made to probe this. Transcriptional analysis and histochemistry revealed that the hypersensitive response (HR) and H_2_O_2_ play critical roles in tea plant defense response to *C. fructicola* [12], and chemical changes of caffeine is considered to be associated with tea–fungi interaction [13]. Nonpathogenic species of *Colletotrichum* was more vulnerable to catechins and caffeine, and differentiation in secondary metabolites might be an important factor leading to the difference in pathogenicity between cultivars [14]. Different communities of *Colletotrichum* with little variability within internal transcribed spacer (ITS) and glyceraldehyde 3-phosphate dehydrogenase (*GAPDH*) regions in the DNA sequence have different virulence [15].

Generally, it was reported that salicylic acid (SA), a secondary metabolite belonging to phenolic compounds, acts as a plant signal or hormone with a regulatory role in a variety of physiological processes under abiotic and biotic stresses, especially as a module in activating disease resistance [16,17,18]. The endogenous SA concentration is related to the conditions of stresses. For instance, SA accumulation, which is responsible for the phenylpropanoid synthesis pathway, was observed in wheat leaves after heavy metal cadmium treatment [19]. In tobacco (*Nicotiana tabacum* L. cv. *Xanthinc*), a transient sharp increase in SA induced by ultraviolet UV light, ozone and mosaic virus parallelly led to pathogenesis-related proteins accumulation and enhanced disease resistance [20]. The *Arabidopsis thaliana* mutants of *sid* and *pad4*, which are involved in synthesis of SA and camalexin (a kind of phytoalexin), were defective in SA synthesis, resulting in them being more susceptible to pathogens [21,22]. The defense compounds including pathogenesis-related proteins (PR-1, PR-2, PR-5), camalexin and H_2_O_2_ could be induced by endogenous SA [22,23,24]. Transgenic plants with salicylate hydroxylase, which converts SA to other compounds, accumulated almost no SA and lost the resistance to virus [25]. When blocked by an inhibitor of phenylpropanoid (precursor of SA) synthesis, the exogenous SA recovered the ability to plant resistance [23]. All these show that SA indeed plays the key role and an important signaling effect in plant resistance, especially in systemic acquired resistance [25,26]. Due to the fact that there has been no study revealing the relationship of SA to the response of tea plant to diseases, it will be interesting to reveal the responses of SA biosynthesis genes and molecular function of SA in the defense response of tea plant to anthracnose infection.

RNA-seq can be focused on assessing the degree of RNA processing and the types of RNA modification, both of which may play an important role in the disease infection process [27,28]. Information of RNA-seq can be used to profile gene expression levels and to reveal the genes involved in plant defense responses [29,30]. RNA-seq analysis needs no reference genome [31] even though tea plant genome data has been available [32]. Developing a transcriptome assembly dataset generating from anthracnose infected tea leaves will be helpful to reveal the molecular defense responses of tea plant to the anthracnose and also to mine molecular markers for MAS used in resistance breeding.

In the present study, transcriptome sequencing by Trinity was performed on the platform of Illumina HiSeq to construct a de novo transcriptome assembly database generated from anthracnose infected tea leaves of two susceptible tea cultivars “Longjing 43” (LJ43) and “Zhenong 139” (ZN139), in which the unigenes were generated and annotated, and important functional genes and metabolic pathways were also revealed. The data sets will provide references for further study on gene expression profiles, biochemical processes and regulation networks associated with tea plant immunity against anthracnose.

## 2. Results

### 2.1. Symptoms of Infected Tea Plants

**Figure 1:**
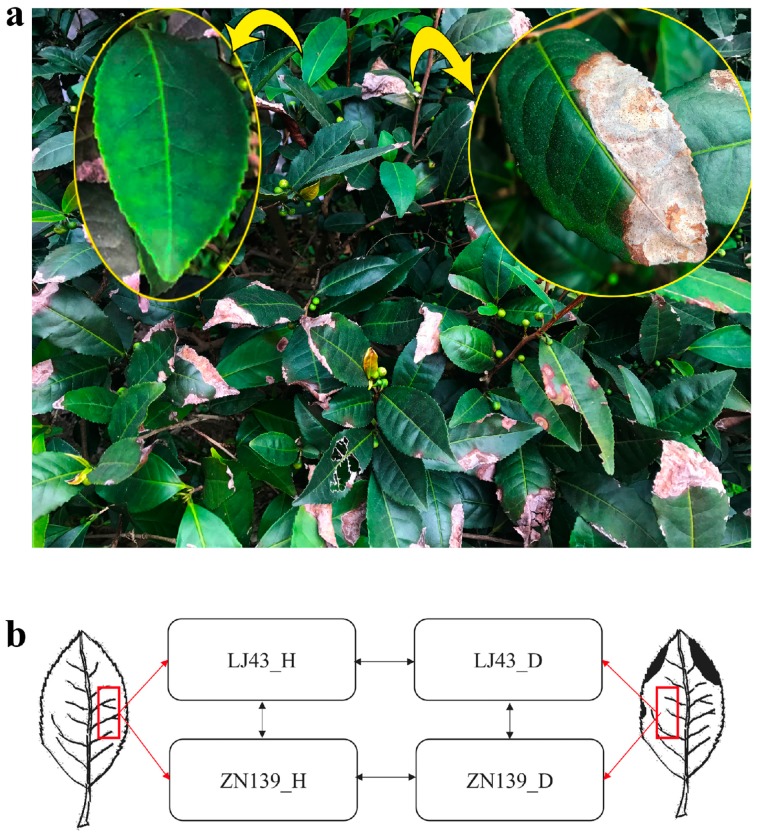
Sample preparation and experiment scheme. (**a**) Anthracnose of tea plant occurrence in tea field. Yellow arrow represents healthy leaf (left) and partially enlarged drawing of anthracnose infected leaf (right). (**b**) A schematic diagram of a healthy leaf (left) and an infected leaf (right). The leaf slices were cut for RNA isolation as described in red boxes. Pairwise comparisons for LJ43 and ZN139 were conducted by arrows. Horizontal arrows mean comparisons between healthy leaves (LJ43_H and ZN139_H) and anthracnose disease infected leaves (LJ43_D & ZN139_D) within cultivar, and the vertical arrow indicates comparisons between cultivars.

We inspected the typical anthracnose symptoms in *Colletotrichum* susceptible tea cultivars “Longjing-43” (LJ43) and “Zhenong-139” (ZN139). The infected leaves were observed to be gray sunken or shrunk necrotic lesions (Figure 1a). In the late stage, the pathogens made the leaves partially withered, fragile, or easily broken, compared to the healthy leaf (Figure 1a left). The infected leaves of both cultivars “LJ43” and “ZN139” showed the same symptoms and the major pathogen isolated from the infected leaves was *Colletotrichum gloeosporioides*. A detailed view of anthracnose-infected tea plant occurrence in tea fields as well as sample preparation and experiment scheme are shown in Figure 1.

### 2.2. Transcriptome Profiling

#### 2.2.1. Sequencing Quality, Assembly Characterization and Functional Annotation

**Figure 2:**
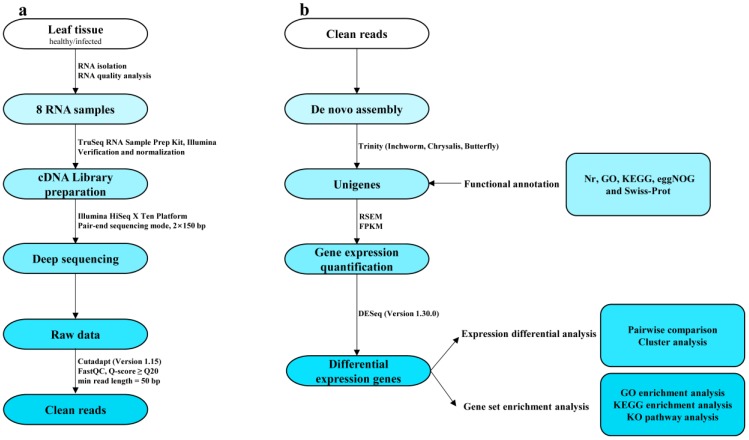
Overview of RNA-seq analysis workflows. (**a**) Acquisition of high-quality sequence (clean reads) from 8 normalized RNA samples. (**b**) The protocol of de novo assembly by Trinity, annotation and downstream analysis for differential expression genes (DEGs).

Transcriptome sequencing was performed using a *de novo* assembly (Figure 2) to reveal molecular resistance or defense information in the host tea plant. Based on Illumina sequencing, we generated 381.52 million pair-end reads encompassed 47.78 billion bases, in total, from the two cultivars with two biological replicants. The percentage of N (ambiguous bases), Q20 (reads with mean error rate <1%), Q30 (reads with mean error rate < 0.1%), GC (guanine-cytosine content) were 0.00%, 95%, 89%, 48.5%, respectively. After quality filtering, about 98% clean reads from the raw reads in each sample were reserved for a de novo assembly (Table 1).

After assembling, 335,186 contigs and 109,316 unigenes were generated from cultivar LJ43, while 352,038 contigs and 115,953 unigenes were generated from cultivar ZN139. Details of total length, mean length, N50 (length-weighted median, i.e., 50% contig length), N50 sequence No. (the number of sequences longer than N50), N90 (length-weighted 90% contig length) and N90 sequence No. (the number of sequences longer than N90) were listed in Table 2.

All unigene sequences were aligned against the five databases, i.e., GO (Gene Ontology), KEGG (Kyoto Encyclopedia of Genes and Genomes), Nr (NCBI non-redundant protein sequences), eggNOG (evolutionary genealogy of genes: Non-supervised Orthologous Groups) and Swiss-prot (Table 2). In cultivar LJ43, 45,230 unigenes (41.4%) were annotated by Nr, 24,892 unigenes (22.8%) by GO, 5799 unigenes (5.3%) by KEGG, 43,025 unigenes (39.4%) by eggNOG and 33,895 unigenes (31.0%) by Swiss-prot, while in cultivar ZN139, 46,383 unigenes (40.0%) were annotated by Nr, 25,168 unigenes (21.7%) by GO, 5873 unigenes (5.1%) by KEGG, 44,067 unigenes (38.0%) by eggNOG and 34,860 unigenes (30.1%) by Swiss-prot. A total of 4605 unigenes (4.21%) and 4677 unigenes (4.03%) in two cultivars were annotated in all five reference databases.

Homology searching against the Nr database gave the explanations containing species distribution of the top hits, *E*-value distribution and similarity distribution (Figure S1). Importantly, about 20.3% unigenes shared the highest homologies to *Coffea canephora* genes. In the eggNOG analysis, annotated unigenes were classified into 26 items with similar descriptions in both cultivars LJ43 (Figure S2a) and ZN139 (Figure S2b). Based on GO classification, all unigenes were classified to three main categories, i.e., molecular function, cellular component and biological process (Table S1). Catalytic activity (GO: 0003824), binding (GO: 0005488), transporter activity (GO: 0005215) and structural molecule activity (GO: 0005198) were the vital subcategories under the category of molecular function. As for cellular component, cell (GO: 0005623), cell part (GO: 0044464), membrane (GO: 0016020), organelle (GO: 0043226) and membrane part (GO: 0044425) were the primary subcategories. With the respect to biological process category, the top five subcategories were metabolic process (GO: 0008152), cellular process (GO: 0009987), single-organism process (GO: 0044699), biological regulation (GO: 0065007) and localization (GO: 0051179). Moreover, all the annotation results of unigenes for both LJ43 and ZN139 were deposited at the public database Figshare (http://doi.org/10.6084/m9.figshare.7706237.v1, 12 February 2019).

#### 2.2.2. Differential Expression and Enrichment Analysis

**Figure 3:**
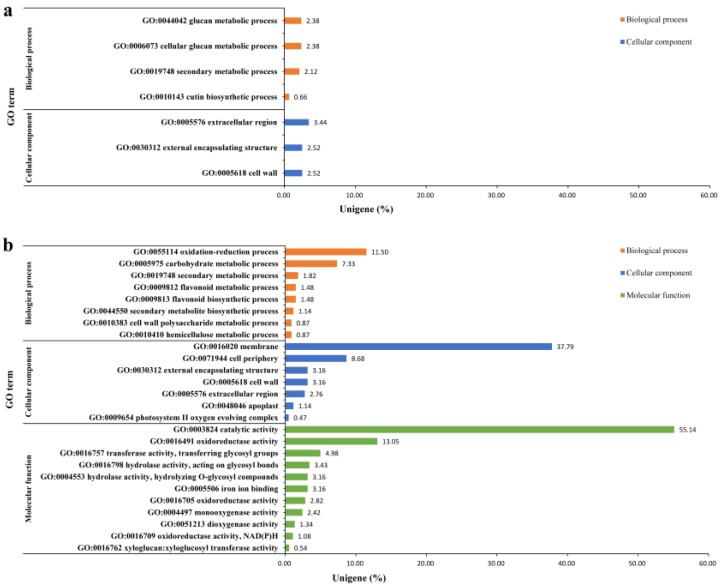
Gene Ontology (GO) enrichment test of cultivars LJ43 (**a**) and ZN139 (**b**). GO terms are divided into three catalogs, i.e., molecular function (green bars), biological processes (orange bars), cellular component (blue bars), which is shown in *Y*-axis. *X*-axis shows the percentage of unigenes in all DEGs aligned to the GO database with Corrected *p*-value (*p*) < 0.05.

Differential expression genes (DEGs) in both cultivars were calculated through anthracnose-infected leaves versus healthy leaves. As shown in Table 3, 1621 DEGs were found in infected leaves of cultivar LJ43, with 1082 up-regulated and 539 down-regulated, while 3089 DEGs were found in infected leaves of cultivar ZN139, with 1527 up-regulated and 1562 down-regulated, compared to the healthy leaves; 755 and 1487 unigenes of the transcripts with GO IDs were mapped by Blast2GO, respectively. Among the DEGs with *p* < 0.05, 7 were annotated by GO, with 4 for biological process and 3 for cellular component in cultivar LJ43 (Figure 3a), while 26 were annotated by GO, with 8 for the biological process, 7 for the cellular component and 11 for the molecular function in cultivar ZN139 (Figure 3b). It was found that catalytic activity, membrane, oxidation-reduction process, cell periphery and carbohydrate metabolic process were strongly responsible to the infection of *Colletotrichum*. Other cellular component categories including the extracellular region, cell wall and external encapsulating structure, as well as the secondary metabolic process were next enriched.

Of course, there are also questions to be explained in the present study. As shown in Figure 3, there was great difference in the GO enrichment between cultivars LJ43 and ZN139, but the exact details leading to the difference remains unknown. It was reported that caffeine and catechins such as (−)-epigallocatechin gallate and (+)-catechin may be involved in the resistance of tea plants to anthracnose [13]. LJ43 has higher level of polyphenols than ZN139 although there are no differences in amino acids and caffeine [33]. Difference in tea polyphenols might be one of the many factors leading to difference in GO enrichment between the two cultivars. Differences in gene abundance might also be partially responsible for the difference in GO enrichment.

**Figure 4:**
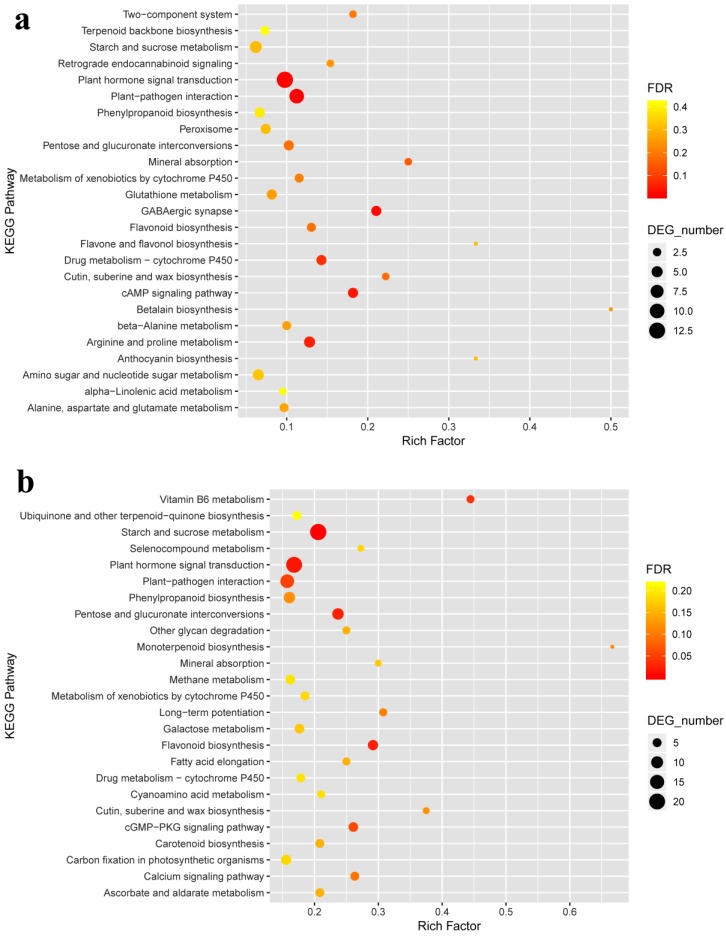
Kyoto Encyclopedia of Genes and Genomes (KEGG) pathway enrichment bubble chart of LJ43 (**a**) and ZN139 (**b**). The Top 25 of pathway enrichment was revealed with low false discovery rate (FDR) (in red colour, significantly) and high FDR (in green colour, insignificantly). *X*-axis shows the percentage of DEGs in total number of genes involved in corresponding KEGG pathway. *Y*-axis expresses KEGG pathway. As well, the size of bubble means the number of DEGs in this item.

As we focused on the top 25 KEGG pathways (Figure 4), it was found that the DEGs were enriched in the pathways involving in plant hormone signal transduction (*ko04075*), plant-pathogen interaction (*ko04626*), starch and sucrose metabolism (*ko00500*) and mineral absorption (*ko04978*) in both cultivars. Besides, flavonoid biosynthesis (*ko00941*), phenylpropanoid biosynthesis (*ko00940*), pentose and glucuronate interconversions (*ko00040*), cutin, suberine and wax (*ko00073*), biosynthesis metabolism of xenobiotics by cytochrome P450 (*ko00980*), and drug metabolism-cytochrome P450 (*ko00982*) also responded to the anthracnose infection (Figure 4).

The differential expression and enrichment analysis results were deposited at the public database Figshare (http://doi.org/10.6084/m9.figshare.7706237.v1, 12 February 2019).

#### 2.2.3. Visualization of Two Vital Plant Metabolic Pathways

**Figure 5:**
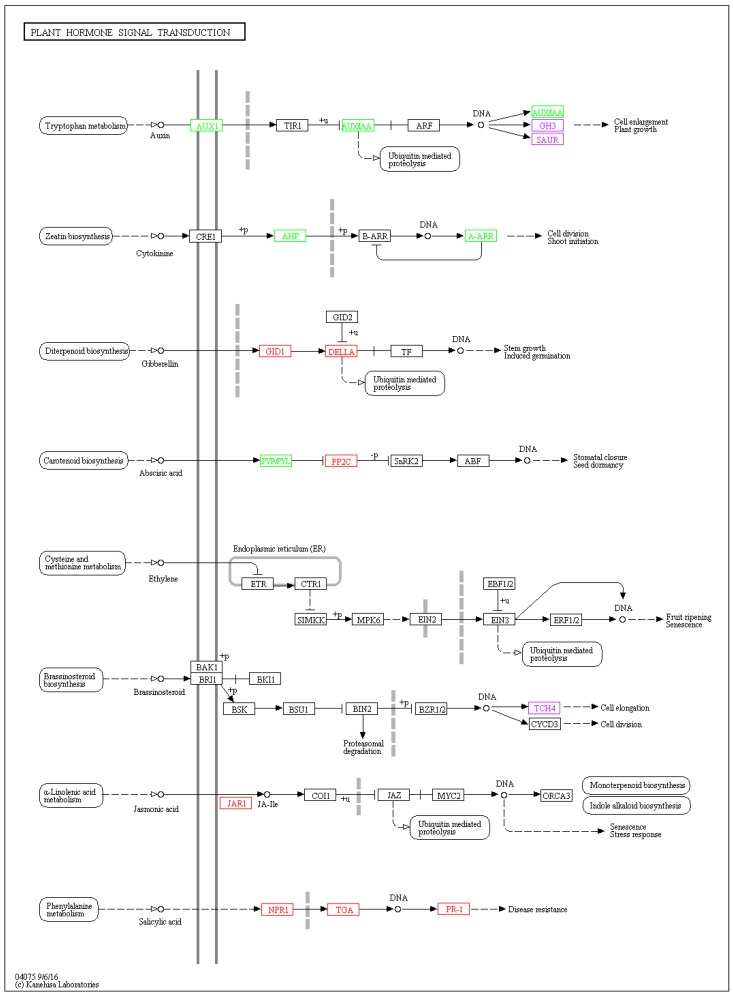
Visualization of plant hormone signal transduction (ko04075) KEGG pathway for two tea cultivars. The DEGs comparisons of anthracnose infected leaves versus healthy leaves was depicted by boxes in different colors, with red colour representing upregulation, green colour representing downregulation and purple colour representing mixture of upregulation and downregulation. Other boxes mean no DEGs mapping to KEGG ontology terms.

Pant hormone signal transduction (*ko04075*, Figure 5) and plant-pathogen interaction (*ko04626*, Figure S3) were vital plant metabolic pathways involved in anthracnose infected process. The nomenclature of gene was based on the entry name from KEGG in this paper. The DEGs mapped to these two pathways showed a same trend in both tested tea cultivars. When the two KEGG pathway results were integrated in one figure (Figure 5), it shows that the genes involving in salicylic acid signal transduction pathway, including *NPR1* (nonexpressor of pathogenesis-related gene 1), *TGA* (TGACG motif-binding factor) and pathogenesis-related protein 1 (*PR1*) were upregulated significantly. *PR1* is directly linked to disease resistance. Some plant hormones with signal transduction components, such as auxin (AUX1, AUX/IAA, GH3, SAUR), cytokinine (AHP, A-ARR), gibberellin (GID1, DELLA), jasmonic acid (JAR1) also play an important role in cell enlargement and division, plant growth, induced germination and stress response. As mentioned in pathogen-associated molecular patterns (PAMP) -triggered immunity, RBOH (respiratory burst oxidase), CALM/CLM (calmodulin), WRKY 25/33 and PR1 were found to be upregulated in the anthracnose infected tea leaves. Upstream CNGC (cyclic nucleotide gated channel) involved in calcium ion transferring was downregulated. The changes of gene expression level might be related to HR, cell wall reinforcement, stomatal closure and defense-related gene induction. The expression level of R genes (*RPM1, PBS1, EDS1*) was upregulated, while the downstream HSP90 (heat shock protein 90kDa beta) was downregulated in effector-triggered immunity.

It is reported that system acquired resistance (SAR) is dependent on salicylic acid signaling and systemic expression of *PR* genes. Immune system resistance (ISR) depends on ethylene and jasmonic acid (JA) but is not associated with the expression of the *PR* genes. Both SAR and ISR do result in broad spectrum resistance. Although an antagonistic interaction between SA and JA pathways was revealed, synergistic interactions were also observed [34]. It is considered that there may be a link between JAR1 and SA-related genes. We searched using key words of brassinosteroids, spermine and heat shock transcription factor in the datasets and found that only heat shock transcription factor (HSP90, being down-regulated by infection) contributed to protect from pathogen infection (Figure 5).

### 2.3. Quantitative Reverse Transcriptase Polymerase Chain Reaction (qRT-PCR) Validation of Salicylic Acid Signaling Related Genes

**Figure 6:**
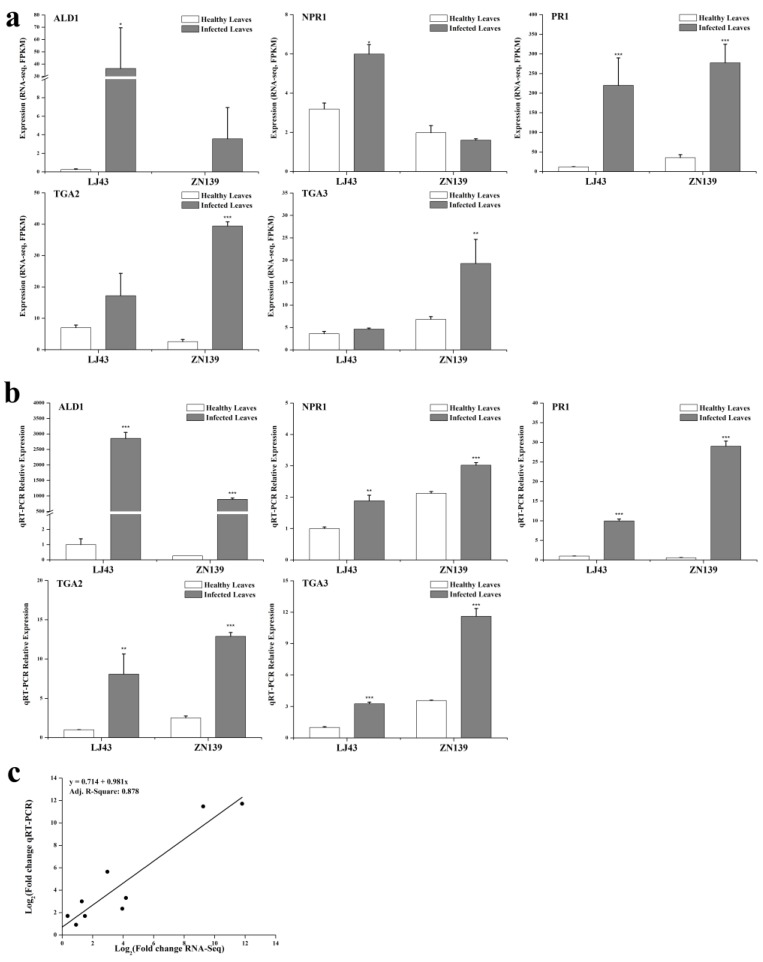
Quantitative reverse transcriptase polymerase chain reaction (qRT-PCR) validation of DEGs and correlation analysis of results between RNA-seq and qRT-PCR. (**a**) FPKM (expected number of Fragments Per Kilobase of transcript sequence per Millions base pairs sequenced) values present RNA-seq expression of 5 DEGs. The sequence of *ALD1* could be found in unigene ID LJ62765_g1 and ZN66762_g1, as *NPR1* in LJ68588_g3 and ZN72573_g8, *TGA2* in LJ58444_g1 and ZN66591_g2, *TGA3* in LJ61836_g1 and ZN62926_g1, *PR1* in LJ62639_g1 and ZN62871_g1. All RNA-seq experiments were performed in two biological replicates (*n* = 2), statistical significance *p* of which was generated from DEseq software. (**b**) The relative expression values were firstly normalized using actin as an internal reference, and then made relative to LJ43_H in which the expression value of LJ43_H was fixed as 1. All qRT-PCR experiments were performed in triplicate (*n* = 3). (**c**) Correlation analysis of results between RNA-seq and qRT-PCR. FPKM values from RNA-seq were compared to relative expression levels detected by qRT-PCR. Data are represented as mean ± standard deviation (SD). Compared with related healthy leaves, * means *p* < 0 05, ** means *p* < 0 01 and ***means *p* < 0 001.

To further verify the sequencing results, expression levels of genes involving in salicylic acid signal transduction pathway in healthy leaves and anthracnose infected leaves of cultivars LJ43 and ZN139 were tested by qRT-PCR (quantitative reverse transcriptase polymerase chain reaction). Genes *ALD1* (AGD2-like defense response gene 1, KEGG Orthology K10206), *TGA2* and *TGA3* (K14431), *PR1* (K13449) in the anthracnose infected leaves showed significant higher expression level than healthy leaves (Figure 6). The expression of *NPR1* (K14508) differentiated between cultivar ZN139 and cultivar LJ43, with upregulation in anthracnose infected leaves of LJ43 but no significant difference between healthy leaves (ZN139_H) and infected leaves (ZN139_D) of cultivar ZN139. *NPR1* is constitutively expressed in many plants and is activated by modification after infection rather than at the transcriptional level. However, our transcriptome dataset shows that the gene encoding predicted regulatory protein NPR3 isoform X1 (LJ69756_g5) was significantly upregulated in the infected leaves, but it was not found in SA pathway. The reason why NPR1 and NPR3 were upregulated during the anthracnose infection remains to be investigated.

Correlation analysis confirmed that relative expression values generated by the two methods were consistent, with a linear correlation coefficient R-square of 0.878 (Figure 6c), suggesting that endogenous salicylic acid is involved in the response to anthracnose in the tea plant.

### 2.4. Salicylic Acid Content

**Figure 7:**
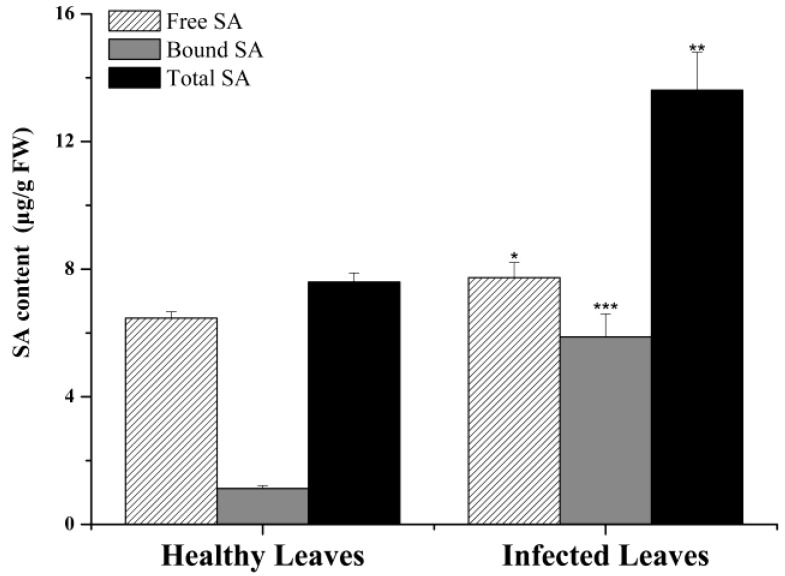
Salicylic acid (SA) content determined by high-performance liquid chromatography (HPLC) in LJ43. All HPLC experiments were performed in triplicate (*n* = 3). Data are represented as means ± SD. Compared with related healthy leaves, * means *p* < 0.05, ** means *p* < 0.01 and *** means *p* < 0.001.

Concentrations of free salicylic acid (SA) and bound SA (the inactive storage form 2-O-β-d-glucosylsalicylic acid) [35] were determined by high-performance liquid chromatography (HPLC) (Figure 7) and it showed that levels of free SA, bound SA and total SA in the anthracnose infected leaves were significantly higher than healthy leaves, suggesting that salicylic acid is an important signaling agent responding to anthracnose infection in tea plant.

## 3. Discussion

Profiling plant immune systems and the pathogen molecules as well as their interaction will provide extraordinary insights into molecular recognition and cell biology of plants [36]. Next-generation molecular approaches such as transcriptomics, metabolomics, whole-genome sequencing and proteomics can be used for this purpose [37]. In the present study, a systematic and accurate transcriptome of healthy leaves and anthracnose-infected leaves of two susceptible tea cultivars was profiled, whose dataset will be of significance for further revealing molecular mechanism of tea plant resistance to anthracnose and mining molecular markers for MAS in tea plant breeding.

Host gene expression fluctuates after pathogen infection and the timing of sampling is important for transcriptome study. However, there have been no studies focused on the expression fluctuation of host genes after pathogen infection. Field investigation showed that the latent period after inoculation of anthracnose pathogens was about 2 weeks [38]. Laboratory tests showed that obvious symptom cold be observed and the resistance capacities of various tea cultivars could be identified after two weeks of wound-inoculation of anthracnose pathogens [8]. In Hangzhou, where we sampled the leaves, the rainy season occurs in June, with humid weather during which anthracnose infection usually takes place. The symptom is observed in early July and so we sampled in early July. Also, tea is a self-sterile plant and all tea cultivars we are planting are cross-pollinated hybrids with a genetically complex background. It would be ideal to obtain a resistant mutant from a disease susceptible tea cultivar. Unfortunately, we have not got one. If a gene showed a same response trend to the infection, it is more definite to conclude that the response has the generality among the susceptible cultivars and is more likely to be related to anthracnose infection when two susceptible cultivars were chosen to be tested.

The present transcriptomic profiling revealed that the oxidation-reduction, catalytic activity, cell wall, membrane and carbohydrate metabolic process in GO analysis (Figure 3), as well as the pathways of Ca^2+^ signaling and SA signaling in KEGG analysis, were strongly responsible to anthracnose infection. SA signaling pathways with remarkable downstream defense gene expression are validated by qRT-PCR in this paper.

Catalytic activity and oxidation-reduction commonly exist in many biological processes or reactions. Most plant cells generate reactive oxygen species (ROS) [39], including hydrogen peroxide, singlet oxygen and hydroxyl radical [40] during their interaction with pathogens, for example *Phytophthora infestans* [41] and *Colletotrichum lindemuthianum* [42]. The generation and accumulation of hydrogen peroxide with a membrane-bound NADPH (nicotinamide adenine dinucleotide phosphate) oxidase is a symptom of the oxidative burst [43], which coincides with programmed cell death (PCD) during HR. The ROS intermediates redox signaling and oxidation-reduction in host plant resistance [44]. Previous study also showed that hydrogen peroxide and HR played a crucial role in tea plant defense [12].

The modification and reinforcement of the cell wall (RCW) as well as deposition of papillae containing callose are considered to be a structural barrier, which can block the penetration of fungal pathogens [42,45]. The detection of hydrogen peroxide at subcellular levels showed that the cross-linking between papillae and HR cells help the pathogen arrest by reinforcing the cell wall apposition [46]. A genetic network of cell-wall damage, regulated by lignin biosynthesis, was characterized in *Arabidopsis thaliana (Col)* [47]. Plants may have evolved a system of dynamic cell wall remodeling to prevent diseases [48]. Many researches elucidated that the changing of cell wall is a way to hinder pathogens entering the plant cells. Hence, we speculated that the cell wall and callose, with complex chemical structure and physiological function, have direct antifungal effects on facilitating tea plant defense.

KEGG analysis revealed that many DEGs were associated with several pathways, particularly in plant hormone signal transduction (Figure 5) and plant-pathogen interaction (Figure S3) in both cultivars. As shown in Figure 4 and Figure S3, a defense-related gene, HR and PCD could be induced through pathogen-associated molecular patterns triggered immunity (PTI) and effector-triggered immunity (ETI) [36]. Ca^2+^ is a fateful factor in PTI, resulting in three regulating pathways. One is the ROS-related pathway, in which upstream RBOH with NADPH oxidases [49] is enhanced highly in anthracnose-infected leaves. The generation of ROS acts as a signal to induce HR and RCW. Ca^2+^ has been reported to activate RBOHs, which is directly phosphorylated responsible to PAMPs perception, at the region of cytosolic N-terminal [50,51]. Ectopic and heterologous expression in the potato (*Solanum tuberosum* cv Rishiri) suggests that calcium-dependent protein kinases 5 regulates the ROS production via St RBOHB (RBOH) [52]. Another is the calmodulin-related pathway. The expression of calmodulin may result in RCW and stomatal closure. A study on barley (*mlo* mutations) against mildew showed that plant defense was regulated by Ca^2+^ dependent interaction with calmodulin and mildew resistance locus O protein, a modulator of plant defense and cell death, in early signaling cascades [53]. Calmodulin detection by enzyme-linked immunosorbent assay (ELISA) showed that calmodulin promoted cell wall regeneration and cell division [54]. The third pathway is related to WRKY transcription factor 25/33, whose high expression induces accumulation of downstream defense proteins encoded by gene PR1. The present results demonstrate that Ca^2+^ is a key component during plant-pathogen interactions, like that described in a previous study [55].

As for ETI in Figure S3, specific disease resistance genes (R) recognize the effectors that enable pathogens overcoming PTI and ETI [36]. When tea plant cell comes into contact with effectors delivered by fungus, the expression level of nucleotide binding leucine rich repeat domains (NB-LRR) proteins [56] encoded by R genes (*RPM1, PBS1, EDS1*) increased markedly, resulting in activation of ETI with a threshold of HR, PCD and defense amplification. In contrast, heat shock protein 90 (HSP90) was down-regulated, which was perhaps related to RPM1 function [57]. A study on heat-shock treatment reported that inhibition of HSP90 caused enhancement of disease resistance by released heat shock transcription factor 1 in tomato cultivar *Natsunokoma* [58], which is consistent with our findings.

**Figure 8:**
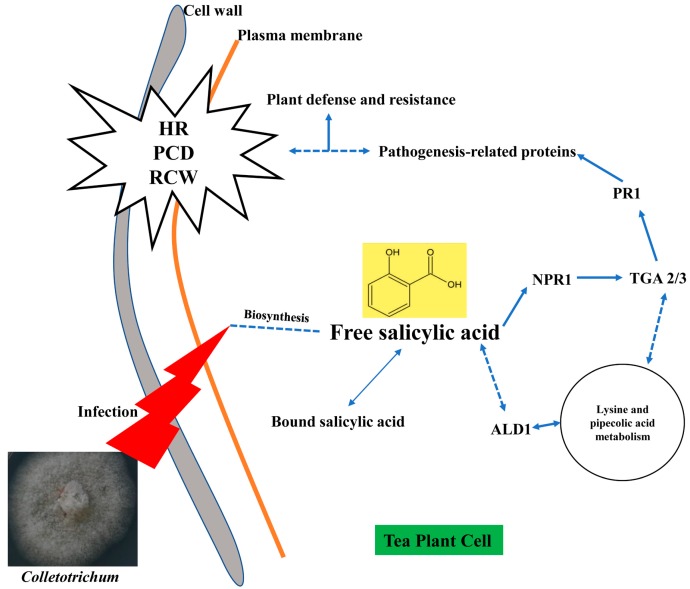
A hypothetical model for SA accumulation and related gene expression in tea plant immunity. Solid lines indicate actual regulations of signaling effect. Dotted lines indicate hypothetical regulations of signaling effect.

It is known that SA, JA and ethylene are three key phytohormones responding to biotic stresses in plant. It is suggested that effective defense against biotrophic or hemibiotrophic pathogens is largely due to PCD in the host, and to associated activation of defense responses regulated by the SA-dependent pathway, while necrotrophic pathogens, benefiting from host cell death, are not limited by SA-dependent defenses, but by JA and ethylene-signaling defense responses [59,60]. Figure 5 shows that the remarkable up-regulation of *NPR1, TGA* and *PR1* genes induced by SA signaling finally facilitates the PR proteins associating with disease resistance to *Colletotrichum*. Based on SA signaling, the effect of *Colletotrichum* on tea leaves is likely to be hemibiotrophic. The qRT-PCR testing confirmed that SA signaling was involved in tea resistance to anthracnose, in which notably high expression levels of *NPR1, TGA2, TGA3* and *PR1* in anthracnose infected leaves were detected in both cultivars (Figure 6). The accumulation of the PR1 protein with antifungal activity enhances plant immunity in the vacuole or extracellular space such as cell wall [26,61]. Among these genes, we demonstrated here that PR1 can be used as a marker for SA-dependent gene expression, being consistent with that in *Arabidopsis* [62]. NPR1 is a critical regulator and receptor, directly binding to SA [63,64] and a test on yeast indicated that the TGA family was a link between NPR1 and PR1 expression [65]. However, another study showed that there were more genes in SA signaling, not limited to those we confirmed before, such as a high affinity SA-binding protein (SABP2) [66]. WRKY factors act as repressors or activators in system acquired resistance (SAR) network, which are induced by SA [67,68]. The present HPLC analysis revealed that the levels of free SA (active form), bound SA (inactive form) and total SA in tea leaves were significantly increased by *Colletotrichum* infection (Figure 7). The results are in accordance with those in *Arabidopsis thaliana (npr1* and *NahG)* showing that antimicrobial proteins encoded by the PR1 superfamily were increased with the increase in endogenous SA levels [69], and also are in accordance with those described in two previous studies on tobacco (*Nicotiana tabacum* L. cv. *Xanthinc*) [20]. The increase in various forms of SA suggests that the free SA is directly involved in tea plant resistance to anthracnose infection and the bound SA might be a stored form of SA, with a potential for defense resistance. In addition, the expression of ALD1, which is involved in the lysine metabolic pathway, was extremely enhanced in the anthracnose infected leaves. The previous study on *Arabidopsis* indicated that ALD1 involving in mediation of plant immunity was more similar to SA-triggered immunity [62]. ALD1-triggered pipecolic acid (PA) and N-hydroxypipecolic acid, whose molecular structures are similar to SA, are largely accumulated in the disease infected plant, where they act as a regulator of SAR activated by SA [70]. The cross-links between SA and PA in tea plant defense need to be further investigated. A hypothetical model was clarified in Figure 8 for SA accumulation and related gene expression involving in the SA signaling pathway in tea immunity.

In conclusion, PR1 and endogenous SA, acting as a key compound, clearly play a pivotal role in defense activation of tea immunity to anthracnose. Fully understanding the pathogen molecule pathways and the interaction between *Colletotrichum* and the tea plant will be helpful to develop agricultural measures to control tea plant anthracnose disease and also to mine molecular markers used in MAS. Despite many recent insights into the tea–*Colletotrichum* interactions by several assays, there is still a large gap in knowledge of mechanisms for further study, such as focuses on mapping to the reference genome, transgenic tea verification and field testing on exogenous SA, SA agonist or SA inhibitor. Furthermore, many other related topics mentioned above, such as cross talks among signaling pathways, other plant hormones, metabolic processes, phytoalexins and etc., need to be verified in future work.

## 4. Materials and Methods

### 4.1. Plant Material

Tea cultivars “Longjing 43” (LJ43) and “Zhenong 139” (ZN139) which are susceptible to anthracnose were used as plant materials in this study. The tea plants were grown in the Experimental Tea Farm of Zhejiang University (Hangzhou, China, 30.3 N, 120.2 E). Leaf samples (10 leaves each sample) were collected in early July 2017 and the leaf slices were cut as described in Figure 1. The responses of a plant to pathogen infections were induced systemically. The healthy leaves were sampled from the individuals which had not completely infected with any part so as to avoid the effects of systematic responses. The leaves were washed with distilled water before cutting the leaf slices to eliminate contamination of RNA from the pathogens and the leaf slices were frozen in liquid nitrogen immediately and then stored at −80 °C until RNA extraction. The healthy leaf samples from LJ43 and ZN139 were labeled as LJ43_H and ZN139_H, and the anthracnose infected leaf samples from LJ43 and ZN139 were labeled as LJ43_D and ZN139_D, respectively (Figure 1). There were two biological replicates for each treatment in the study of de novo transcriptome assembly, and three biological replicates for the studies of qRT-PCR validation and HPLC analysis of salicylic acid.

### 4.2. Total RNA Extraction, cDNA Library Construction and Deep Sequencing

Total RNA was extracted using RNAprep Pure Kit for Polysaccharides and Polyphenolics rich plant (Cat. no. DP441, Tiangen Biotech Co., Ltd., Beijing, China) according to the instructions of the kit. Concentration and quality of the RNA were assessed using Agilent 2100 Bioanalyzer (Agilent Technologies Inc., Santa Clara, USA), and those with 1.8 ≤ OD260 nm/OD280 nm ≤ 2.1 and RIN ≥ 6.5 were used in subsequent tests. RNA integrity was evaluated by agarose gel electrophoresis. Three micrograms of RNA were used as input material for cDNA preparations.

The cDNA library was constructed according to the protocol of TruSeqRNA Sample Prep Kit (Illumina Inco., San Diego, CA, USA). The quality and quantity of libraries were checked by fluorospectrophotometry (Quant-iTPicoGreen dsDNA Assay Kit, Invitrogen, P7589; Quantifluor-ST fluorometer E6090, Promega, CA, USA) and Agilent 2100 Bioanalyzer with Agilent Bioanalyzer High Sensitivity DNA chip Kit (5067-4626, Agilent Technologies Inc., Santa Clara, USA). Each normalized cDNA library (2 nM) was gradually diluted to 4–5 pM and the sequencing was performed on Illumina HiSeq X Ten Platform in 2 × 150 bp pair-end sequencing mode (Figure 2a) and the original data in FASTQ format (Raw Data) was then generated. The quality assessment of raw data in FASTQ format was done with FastQC [71] (http://www.bioinformatics.babraham.ac.uk/projects/fastqc, 18 January 2018, Q-score ≥ Q20, min read length = 50 bp). The connectors were removed by Cutadapt (Version 1.15) and clean reads were prepared for the further analysis (Figure 2a).

### 4.3. De Novo Assembly and Functional Annotation

The present tests were carried out in 2017, when the reference genome of tea plant had not been published. Accordingly, de novo assembly was performed to reconstruct the transcriptomes. We used Trinity (Version r20140717, default k-mer = 25 bp) [72,73], consisting of three software modules: Inchworm, Chrysalis and Butterfly, to efficiently process massive clean reads based on robust reconstruction and analysis of De Bruijn Graph (DBG) [72]. Briefly, clean reads with overlap joints were firstly combined to form longer fragments, i.e., contigs. Related pair-end reads and contigs were clustered to form unigenes. Finally, non-redundant unigenes for functional annotation were obtained [73].

The unigene sequences were compared using the local BLASTX [74] program (*E*-value ≤1e-5) against 5 public databases: Nr [75], GO [76], KEGG [77], Swiss-Prot [78] and eggNOG [79]. GO terms were identified on the platform BLAST2GO [80]. KEGG Pathways and KEGG Orthologs were annotated by KAAS [81] (KEGG Automatic Annotation Server, http://www.genome.jp/tools/kaas/, 18 January 2018).

### 4.4. Differential Expression Genes (DEGs) Analysis

Firstly, the software RSEM [82] was used to compare read count values on each unigene as original expression, and then standardized as FPKM (expected number of fragments per kilobase of transcript sequence per millions of base pairs sequenced). Then DESeq (Version 1.30.0) was used to analyze DEGs [83]. The screen criteria were expression difference multiple |log2 Fold Change| > 1 (*p* < 0.05). The bi-directional clustering analysis of DEGs in different samples was performed using R language. Then we mapped all the DEGs to GO databases to reveal the gene enrichment in each term. Furthermore, the DEGs involved in the KEGG pathways including signaling and metabolic pathways were assigned. All the downstream analysis was described in Figure 2b.

### 4.5. qRT-PCR Validation

To verify the expression levels of DEGs obtained from RNA-seq, qRT-PCR were carried out following the previous protocol [84]. The primary DEGs involved in salicylic acid signal transduction pathway were chosen for validation and specific primers were designed using the PrimerQuest Tool (https://sg.idtdna.com/PrimerQuest/Home/Index, 10 April 2018–23 April 2018). qRT-PCR was run in the machine StepOne Plus (Applied Biosystems, Foster City, CA, USA). The qRT-PCR test was generated using triplicate samples. Each relative expression level was calculated using the 2^−ΔΔCT^ method [85] with *β-actin* gene as internal control.

### 4.6. Salicylic Acid Extraction and High-Performance Liquid Chromatography (HPLC) Quantification

The extraction of total SA including free SA and bound SA was performed following the published method [35,86]. The cut leaf slices stored at −80 °C were pulverized in a mortar with liquid nitrogen condition using a pestle. About 0.1 g homogenized powders was mixed with 1 mL pre-cooling 90% methanol, stood overnight, and centrifuged at 8000× *g* for 10 min. The supernatants were collected while the sediment was re-suspended in 0.5 mL 90% methanol and further extracted for 2 h. The centrifugation and collection were repeated as before. The combined supernatants were evaporated using a SpeedVac machine at 40 °C to 0.3 mL aqueous solution. 20 μL trichloroacetic acid (1 mg/mL) was added to the aqueous solution and mixed by vortex. Partition with 1 mL ethyl acetate: cyclohexane (1:1, *v*/*v*) was carried out twice. The organic phase with free SA was dried by nitrogen blowing and dissolved in 0.5 mL mobile phase for HPLC test. The aqueous phase containing bound SA was acid-hydrolyze by adding 0.5 mL HCl (8 M) to release free SA. The same steps for obtaining free SA were implemented again. At this moment, the bound SA was determined in the form of free SA. The contents of free SA and bound SA in the samples were then determined by an Agilent 1100 HPLC system with fluorescence detection. Chromatographic separation was performed using a Kromasil C18 reverse phase column (5 μm, 250 mm × 4.6 mm). The mobile phase was a mixture of 1% acetic acid aqueous solution with 1% phosphoric acid: methanol (40:60, *v*/*v*). The flow rate was set at 0.8 mL/min for 50 min in total. The column temperature was set at 35 °C and injection volume was 10 μL. Excitation and emission wavelengths were 294 nm and 426 nm, respectively.

The data of qRT-PCR and HPLC were expressed as mean (mean values of three independent experiments) ± SD (standard deviation). An analysis of variance (ANOVA) test, mainly using least significant difference (LSD), was used for measuring statistical significance by means of SPSS and EXCEL software.

## Figures and Tables

**Table 1:** Summary statistics of raw reads and clean reads.

Sample Name	LJ43_H	LJ43_D	ZN139_H	ZN139_D	All Samples
Raw Reads Number (million)	38.86 ± 1.19	42.94 ± 0.03	40.06 ± 3.52	37.40 ± 2.27	318.52
Total Bases (billion bp)	5.83 ± 0.18	6.44 ± 0.00	6.01 ± 0.53	5.61 ± 0.34	47.78
N (%)	0.00 ± 0.00	0.00 ± 0.00	0.00 ± 0.00	0.00 ± 0.00	
Q20 (%)	95.29 ± 0.38	95.43 ± 0.13	95.06 ± 0.24	95.32 ± 0.32	
Q30 (%)	89.38 ± 0.69	89.67 ± 0.27	88.89 ± 0.40	89.47 ± 0.57	
GC (%)	48.72 ± 0.01	49.07 ± 0.13	48.79 ± 0.30	48.41 ± 0.72	
Clean Reads (%)	98.04 ± 0.28	98.11 ± 0.05	97.99 ± 0.17	97.99 ± 0.25	

**Table 2:** Information of assembled contigs, unigenes and annotations for two cultivars.

Cultivar		LJ43	ZN139
Contig	Total Length (bp)	102,521,451	107,758,129
	Sequence Number	335,186	352,038
	Mean Length (bp)	306	306
	N50 (bp)	404	405
	N50 Sequence No.	54,939	58,050
	N90 (bp)	150	150
	N90 Sequence No.	251,498	264,172
Unigene	Total Length (bp)	65,163,329	68,536,138
	Sequence Number	109,316	115,953
	Mean Length (bp)	596	591
	N50 (bp)	821	803
	N50 Sequence No.	20,020	21,541
	N90 (bp)	261	260
	N90 Sequence No.	81,051	86,082
Annotated Unigene Number in Database	Nr	45,230 (41.38%)	46,383 (40%)
	GO	24,892 (22.77%)	25,168 (21.71%)
	KEGG	5799 (5.3%)	5873 (5.06%)
	eggNOG	43,025 (39.36%)	44,067 (38%)
	Swiss-Prot	33,895 (31.01%)	34,860 (30.06%)
	In all database	4605 (4.21%)	4677 (4.03%)

**Table 3:** List of total DEGs in case-control study ^1^.

Control	Case	Up-Regulated DEGs	Down-Regulated DEGs	Total DEGs
LJ43_H	LJ43_D	1082	539	1621
ZN139_H	ZN139_D	1527	1562	3089

^1^ Healthy leaf were identified as control group, and anthracnose infected leaves as case group.

## Supplementary Materials

Supplementary materials can be found at https://www.mdpi.com/1422-0067/20/10/2439/s1. Raw data files generated by RNA-seq were deposited in the NCBI Sequence Read Archive SRP162639. The files of unigenes were deposited at GenBank Transcriptome Shotgun Assembly TSA GHEL00000000. The annotation and differential expression analysis results of the unigenes were deposited at Figshare (http://doi.org/10.6084/m9.figshare.7706237.v1, 12 February 2019). Table S1. All subcategories of GO annotations. Table S2. qRT-PCR primers of selected unigenes. Figure S1. Characteristics of homology searching in *Camellia sinensis* against the Nr database. Figure S2. eggNOG functional classification of consensus sequence. Figure S3. Visualization of plant-pathogen interaction (ko04626) KEGG pathway for two tea cultivars.

## Abbreviations

*ALD1*	AGD2-like defense response gene 1
DBG	De Bruijn Graph
DEGs	Differential expression genes
eggNOG	Evolutionary genealogy of genes: Non-supervised Orthologous Groups
ETI	Effector-triggered immunity
FDR	False discovery rate
FPKM	Expected number of Fragments Per Kilobase of transcript sequence per Millions base pairs sequenced
GO	Gene Ontology
HPLC	High-performance liquid chromatography
HR	Hypersensitive response
ISR	Immune system resistance
ITS	Internal transcribed spacer
KAAS	KEGG Automatic Annotation Server
KEGG	Kyoto Encyclopedia of Genes and Genome
MAS	Molecular assisted selection
*NPR1*	Nonexpressor of pathogenesis-related gene 1
Nr	NCBI non-redundant protein sequences
*p*	*p*-value
PCD	Programmed cell death
*PR1*	Pathogenesis-related gene 1
PTI	Pathogen-associated molecular patterns triggered immunity
qRT-PCR	Quantitative reverse transcriptase polymerase chain reaction
RCW	Reinforcement of the cell wall
ROS	Reactive oxygen species
SA	Salicylic acid
SAR	System acquired resistance
TGA	Trans-activating TGA Factors
