# Supplementary material for: Involvement of Salicylic Acid in Anthracnose Infection in Tea Plants Revealed by Transcriptome Profiling
Source: Int J Mol Sci. 2019 May 17;20(10):2439. doi: 10.3390/ijms20102439 (PMC6566613; doi:10.3390/ijms20102439)
Supplement: Supplementary file 1 [file ijms-20-02439-s001.pdf]

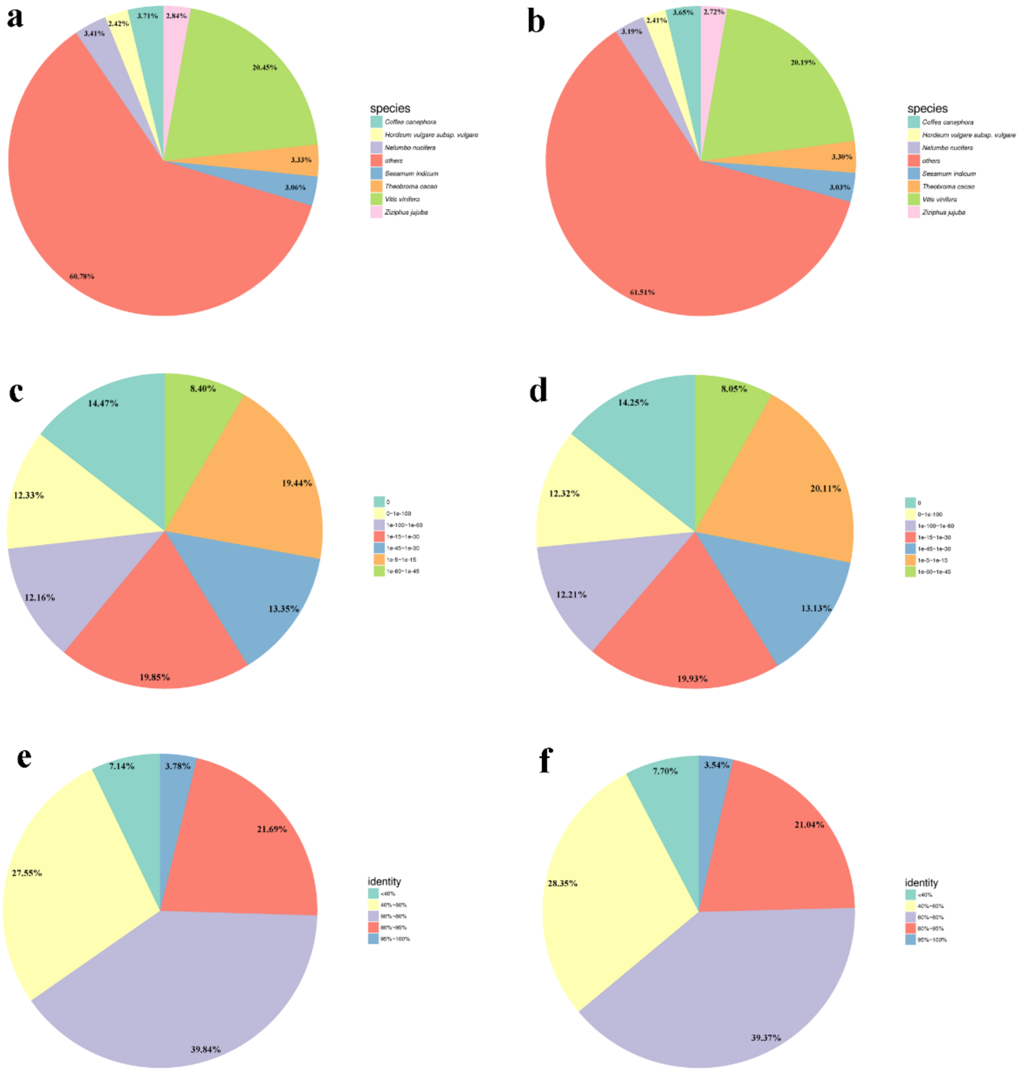

Figure S1. Characteristics of homology searching in Camellia sinensis against the NR database. (a, b) Species distribution of the top hits using BLASTX. (c, d) E-value distribution of NR annotation. (e, f) The similarity distribution of annotation in NR database. a, c, e being from cultivar LJ43, and b, d, f being from cultivar ZN139.

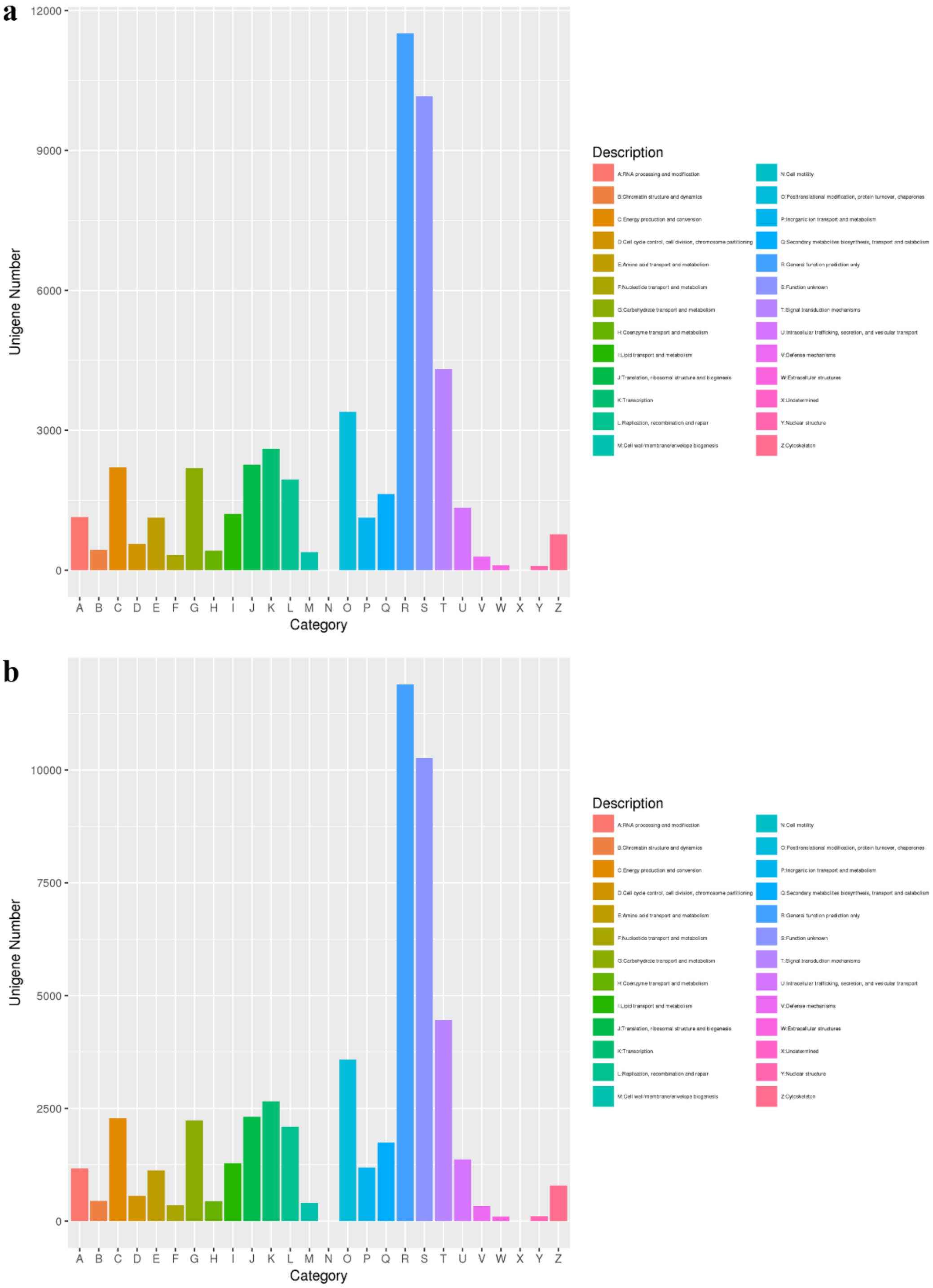

Figure S2. eggNOG functional classification of consensus sequence. (a) LJ43. (b) ZN139.

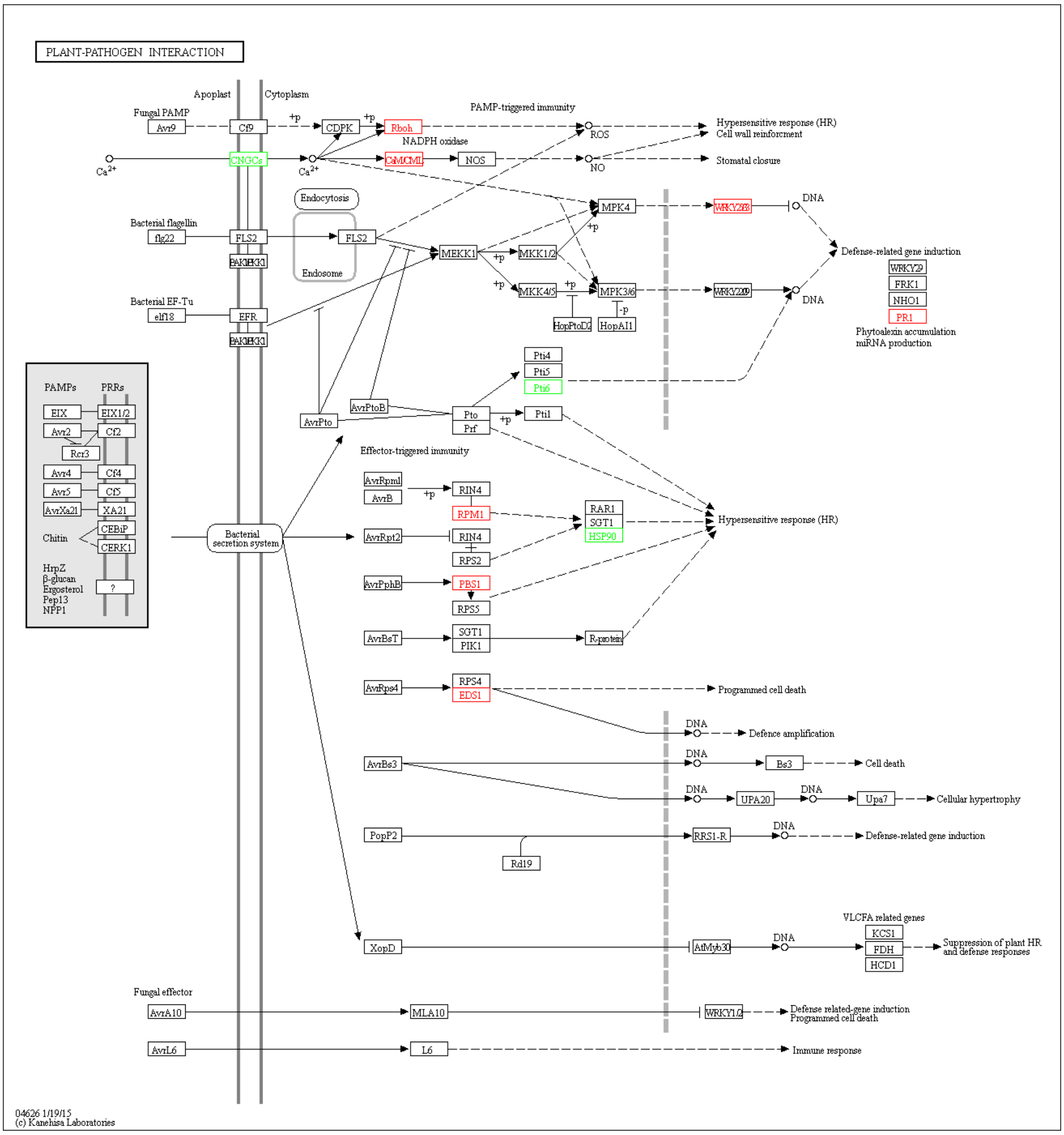

Figure S3. Visualization of plant-pathogen interaction (ko04626) KEGG pathway for two tea cultivars.

Table S1. All subcategories of GO annotations.

LJ43 Catergory	GO	Description	Number
biological_process	GO:0000003	reproduction	389
biological_process	GO:0001906	cell killing	2
biological_process	GO:0002376	immune system process	65
biological_process	GO:0007610	behavior	0
biological_process	GO:0008152	metabolic process	12429
biological_process	GO:0009987	cellular process	12386
biological_process	GO:0022414	reproductive process	389
biological_process	GO:0022610	biological adhesion	3
biological_process	GO:0023052	signaling	720
biological_process	GO:0032501	multicellular organismal process	681

biological_process	GO:0032502	developmental process	702
biological_process	GO:0040007	growth	96
biological_process	GO:0040011	locomotion	5
biological_process	GO:0044699	single-organism process	8182
biological_process	GO:0044848	biological phase	0
biological_process	GO:0048511	rhythmic process	8
biological_process	GO:0050896	response to stimulus	2112
biological_process	GO:0051179	localization	2634
biological_process	GO:0051704	multi-organism process	264
biological_process	GO:0065007	biological regulation	2812
biological_process	GO:0071840	cellular component organization or biogenesis	1753
biological_process	GO:0098743	cell aggregation	0
biological_process	GO:0098754	detoxification	163
biological_process	GO:0099531	presynaptic process involved in synaptic transmission	0
cellular_component	GO:0005576	extracellular region	302
cellular_component	GO:0005623	cell	9376
cellular_component	GO:0009295	nucleoid	18
cellular_component	GO:0016020	membrane	8177
cellular_component	GO:0019012	virion	31
cellular_component	GO:0030054	cell junction	256
cellular_component	GO:0031012	extracellular matrix	3
cellular_component	GO:0031974	membrane-enclosed lumen	516
cellular_component	GO:0032991	macromolecular complex	3002
cellular_component	GO:0039679	viral occlusion body	0
cellular_component	GO:0043226	organelle	6518
cellular_component	GO:0044215	other organism	0
cellular_component	GO:0044217	other organism part	0
cellular_component	GO:0044420	extracellular matrix component	0
cellular_component	GO:0044421	extracellular region part	20
cellular_component	GO:0044422	organelle part	2995
cellular_component	GO:0044423	virion part	31
cellular_component	GO:0044425	membrane part	6130
cellular_component	GO:0044456	synapse part	0
cellular_component	GO:0044464	cell part	9287
cellular_component	GO:0045202	synapse	0
cellular_component	GO:0055044	symplast	256
cellular_component	GO:0097423	mitochondrion-associated adherens complex	0
cellular_component	GO:0099512	supramolecular fiber	174
molecular_function	GO:0000988	transcription factor activity, protein binding	74
molecular_function	GO:0001071	nucleic acid binding transcription factor activity	368
molecular_function	GO:0003824	catalytic activity	12293
molecular_function	GO:0004871	signal transducer activity	201
molecular_function	GO:0005198	structural molecule activity	1224

molecular_function	GO:0005215	transporter activity	1512
molecular_function	GO:0005488	binding	11962
molecular_function	GO:0009055	electron carrier activity	180
molecular_function	GO:0016015	morphogen activity	0
molecular_function	GO:0016209	antioxidant activity	190
molecular_function	GO:0016530	metallochaperone activity	4
molecular_function	GO:0031386	protein tag	5
molecular_function	GO:0036370	D-alanyl carrier activity	0
molecular_function	GO:0042056	chemoattractant activity	0
molecular_function	GO:0045182	translation regulator activity	0
molecular_function	GO:0045499	chemorepellent activity	0
molecular_function	GO:0045735	nutrient reservoir activity	15
molecular_function	GO:0060089	molecular transducer activity	120
molecular_function	GO:0098772	molecular function regulator	248
**ZN139 Catergory**	**GO**	**Description**	**Number**
biological_process	GO:0000003	reproduction	401
biological_process	GO:0001906	cell killing	2
biological_process	GO:0002376	immune system process	53
biological_process	GO:0007610	behavior	0
biological_process	GO:0008152	metabolic process	12554
biological_process	GO:0009987	cellular process	12554
biological_process	GO:0022414	reproductive process	400
biological_process	GO:0022610	biological adhesion	2
biological_process	GO:0023052	signaling	723
biological_process	GO:0032501	multicellular organismal process	651
biological_process	GO:0032502	developmental process	654
biological_process	GO:0040007	growth	109
biological_process	GO:0040011	locomotion	4
biological_process	GO:0044699	single-organism process	8110
biological_process	GO:0044848	biological phase	0
biological_process	GO:0048511	rhythmic process	6
biological_process	GO:0050896	response to stimulus	2105
biological_process	GO:0051179	localization	2638
biological_process	GO:0051704	multi-organism process	258
biological_process	GO:0065007	biological regulation	2770
biological_process	GO:0071840	cellular component organization or biogenesis	1748
biological_process	GO:0098743	cell aggregation	0
biological_process	GO:0098754	detoxification	157
biological_process	GO:0099531	presynaptic process involved in synaptic transmission	0
cellular_component	GO:0005576	extracellular region	294
cellular_component	GO:0005623	cell	9543
cellular_component	GO:0009295	nucleoid	20
cellular_component	GO:0016020	membrane	8193

cellular_component	GO:0019012	virion	36
cellular_component	GO:0030054	cell junction	260
cellular_component	GO:0031012	extracellular matrix	5
cellular_component	GO:0031974	membrane-enclosed lumen	494
cellular_component	GO:0032991	macromolecular complex	3066
cellular_component	GO:0039679	viral occlusion body	0
cellular_component	GO:0043226	organelle	6648
cellular_component	GO:0044215	other organism	0
cellular_component	GO:0044217	other organism part	0
cellular_component	GO:0044420	extracellular matrix component	0
cellular_component	GO:0044421	extracellular region part	22
cellular_component	GO:0044422	organelle part	3046
cellular_component	GO:0044423	virion part	36
cellular_component	GO:0044425	membrane part	6087
cellular_component	GO:0044456	synapse part	0
cellular_component	GO:0044464	cell part	9464
cellular_component	GO:0045202	synapse	0
cellular_component	GO:0055044	symplast	260
cellular_component	GO:0097423	mitochondrion-associated adherens complex	0
cellular_component	GO:0099512	supramolecular fiber	150
molecular_function	GO:0000988	transcription factor activity, protein binding	73
molecular_function	GO:0001071	nucleic acid binding transcription factor activity	374
molecular_function	GO:0003824	catalytic activity	12431
molecular_function	GO:0004871	signal transducer activity	194
molecular_function	GO:0005198	structural molecule activity	1281
molecular_function	GO:0005215	transporter activity	1503
molecular_function	GO:0005488	binding	12049
molecular_function	GO:0009055	electron carrier activity	173
molecular_function	GO:0016015	morphogen activity	0
molecular_function	GO:0016209	antioxidant activity	188
molecular_function	GO:0016530	metallochaperone activity	4
molecular_function	GO:0031386	protein tag	7
molecular_function	GO:0036370	D-alanyl carrier activity	0
molecular_function	GO:0042056	chemoattractant activity	0
molecular_function	GO:0045182	translation regulator activity	0
molecular_function	GO:0045499	chemorepellent activity	0
molecular_function	GO:0045735	nutrient reservoir activity	19
molecular_function	GO:0060089	molecular transducer activity	111
molecular_function	GO:0098772	molecular function regulator	258

Table S2. qRT-PCR primers of selected unigenes.

Gene ID	Primer Sequence	
actin	Forward Primer	CTTCCTCATGCTATCCTCCGTCTT
	Reverse Primer	ATTTCCCGTTCAGCAGTGGTG
ALD1	Forward Primer	GCAAGGCAACATGGAATTAAGG
	Reverse Primer	GAGCATCTGAAGGCGAGATATG
NPR1-F	Forward Primer	CGGTGAATTCAGTGGGATATGA
	Reverse Primer	TGCTCTCTCGCCACAATTAG
TGA3-F	Forward Primer	GAAAGCAGATGCTGCAAAGG
	Reverse Primer	GGAATCCTCCAAGCCAAAGA
TGA2-F	Forward Primer	CCTGCATGGCCTATGAGATT
	Reverse Primer	CAGATGTGCCTCAACCTTACT
PR1-F	Forward Primer	TGTCGGATGCAGTGAAGATG
	Reverse Primer	ACCCTATTTGTGCGGTGTT
